# Distinct roles of α- and βCaMKII in controlling long-term potentiation of GABA_A_-receptor mediated transmission in murine Purkinje cells

**DOI:** 10.3389/fncel.2014.00016

**Published:** 2014-02-03

**Authors:** Zhenyu Gao, Geeske M. van Woerden, Ype Elgersma, Chris I. De Zeeuw, Freek E. Hoebeek

**Affiliations:** ^1^Department of Neuroscience, Erasmus Medical CentreRotterdam, Netherlands; ^2^Netherlands Institute for Neuroscience, Royal Academy of Arts and SciencesAmsterdam, Netherlands

**Keywords:** CaMKII, plasticity, GABAAR

## Abstract

Calcium/Calmodulin-dependent kinase type II (CaMKII) is essential for various forms of synaptic plasticity. The predominant α- and βCaMKII isoforms have both been shown to contribute to specific forms of plasticity at excitatory synapses, but little is known about their functions at inhibitory synapses. Here we investigated the role of both isoforms in long-term potentiation of the inhibitory molecular layer interneuron to Purkinje cell synapse (MLI-PC iLTP) upon climbing fiber (CF) stimulation. We demonstrate that deleting either the α- or βCaMKII isoform affected MLI-PC iLTP. In the presence of the PP2B blocker cyclosporin A, CF stimulation elicited iLTP in *Camk2b*^-^^/^^-^ mice, but not in *Camk2a*^-^^/^^-^ mice. Moreover, co-activation of the MLIs and CF suppressed iLTP in wild-type mice through activation of GABA_B_-receptors, whereas it evoked iLTP in *Camk2b*^-^^/^^-^. This reversal of the effect of αCaMKII activity in *Camk2b*^-^^/^^-^ mutants upon co-activation did not critically involve protein kinase A, but depended on calcium release from internal stores. Our results indicate that α- and βCaMKII isoforms in Purkinje cells can be differentially activated and serve distinct roles in controlling iLTP. We propose that the CaMKII holo-enzyme may be selectively activated by various GABA_B_-mediated pathways and that the presence of the βCaMKII isoform determines their impact on inhibitory plasticity.

## INTRODUCTION

Calcium/Calmodulin-dependent Kinase type II (CaMKII) is one of the most densely expressed proteins in the central nervous system (Erondu and Kennedy, 1985). The intracellular signaling pathways that are controlled by CaMKII have been shown to be important for memory formation by controlling synaptic plasticity (Silva et al., 1992a, 1992b; Colbran and Brown, 2004; Wayman et al., 2008). The CaMKII holo-enzyme is essential for pre- and post-synaptic mechanisms at both excitatory and inhibitory synapses in hippocampal, amygdalar, cortical, and cerebellar neurons (Castillo et al., 2011), which highlights the importance of this molecule for proper neuronal functioning.

In the brain the CaMKII holo-enzyme comprises predominantly α- and βCaMKII subunits (Miller and Kennedy, 1985). βCaMKII differs from αCaMKII by its actin binding domain and higher calcium sensitivity (Shen et al., 1998; Brocke et al., 1999; Thiagarajan et al., 2002; Fink et al., 2003; Cho et al., 2007). Recent studies revealed that each isoform has a distinct function in controlling synaptic plasticity at excitatory synapses in the neurons that express both α- and βCaMKII. For instance, deletion of αCaMKII results in disrupted long-term depression (LTD) at the excitatory granule cell – Purkinje cell synapse, whereas the deletion of βCaMKII bidirectionally reverses LTD and long-term potentiation (LTP; Hansel et al., 2006; van Woerden et al., 2009).

The molecular mechanisms that underlie long-term plasticity at inhibitory and excitatory synapses show extensive overlap, but it remains to be elucidated whether α- and βCaMKII serve distinct functions in controlling plasticity at inhibitory synapses. The functional relevance of this form of plasticity for cerebellar learning has been previously predicted (Wulff et al., 2009; Gao et al., 2012). Indeed, Tanaka et al. (2013) recently showed that it is involved in adaptation of the vestibulo-ocular reflex, which is controlled by the flocculus of the cerebellum. Here we studied the impact of genetic ablation of αCaMKII or βCaMKII on the expression of synaptic plasticity at the inhibitory molecular layer interneuron – Purkinje cell (MLI-PC) synapses using *Camk2a*^-^^/^^-^ and *Camk2b*^-^^/^^-^ mutant mice. Our results show that α- and βCaMKII isoforms serve distinct roles in controlling LTP at this inhibitory synapse (iLTP).

## MATERIAL AND METHODS

### ETHICAL APPROVAL

All studies were performed in accordance with the guidelines for animal experiments of the Erasmus Medical Center and the Dutch national legislation. All experiments and analyses were performed by scientists blinded to the genotype of the mouse.

### ANIMALS

*Camk2a*^-^^/^^-^ mice were generated as previously described (Elgersma et al., 2002) and for *Camk2b*^-^^/^^-^ we used exon 2 knock-out mice, which showed complete loss of βCaMKII expression and ataxia, as described previously for the *Camk2b* exon 11 knock-out (van Woerden et al., 2009). Homozygous mice and wt littermates (both genders; generated by heterozygous × heterozygous breeding) ranging from postnatal day (P) 17–21 were used in all experiments. Animals were maintained at 22 ± 2^°^C with 12 h dark and light cycle and were provided with food and water *ad libitum*.

### SLICE PREPARATION FOR ELECTROPHYSIOLOGY

*Camk2a*^-^^/^^-^ and *Camk2b*^-^^/^^-^ mice and wt littermates were decapitated under isoflurane anesthesia. Subsequently, the cerebellum was removed and transferred into ice-cold slicing medium that contains (in mM): 240 Sucrose, 5 KCl, 1.25 Na_2_HPO_4_ 2 MgSO_4_, 1 CaCl_2_, 26 NaHCO_3_, and 10 D-Glucose, bubbled with 95% O_2_ and 5% CO_2_. Parasagittal slices (250 μm thick) of the cerebellar vermis were cut using a vibratome (VT1000S, Leica) and kept in ACSF containing (in mM): 124 NaCl, 5 KCl, 1.25 Na_2_HPO_4_, 2 MgSO_4_, 2 CaCl_2_, 26 NaHCO_3_, and 20 D-Glucose, bubbled with 95% O_2_ and 5% CO_2_ for >1 h at 34 ± 1^°^C before the experiments started.

### WHOLE-CELL ELECTROPHYSIOLOGY

Experiments were performed with a constant flow of oxygenated ACSF (1.5–2.0 ml/min). Purkinje cells were visualized using an upright microscope (Axioskop 2 FS plus, Carl Zeiss, Germany) equipped with a 40X water immersion objective. Patch-clamp recordings were performed using an EPC-10 double amplifier (HEKA electronics, Lambrecht, Germany). All recordings were performed at 34 ± 1^°^C.

Whole cell current clamp recordings of Purkinje cells were performed using borosilicate pipettes (*R*_pip_ = 2–4 Ω) filled with intracellular solution containing (in mM): 130 K-Gluconate, 10 KOH, 3.48 MgCl_2_, 4 NaCl, 10 HEPES, 4 Na_2_ATP, 0.4 Na_3_GTP, and 17.5 sucrose (pH 7.25, osmolarity 295). GABAergic MLI-PC synapses were stimulated as previously described (Mittmann and Häusser, 2007). In short, one patching pipette filled with ACSF was located at the molecular layer >200 μm lateral from Purkinje cells to avoid activating parallel fiber-Purkinje cell synapses. Our conditions resulted in a reversal potential for IPSPs of -75 to -78 mV with corrected liquid junction potentials. IPSPs were completely blocked by bath-applied non-competitive GABA_A_-receptor blockers picrotoxin (100 μM) or SR95531 (10 μM). Evoked IPSPs from MLI-PC synapses appeared to be all or none, suggesting direct stimulations at stellate cell somata. To avoid intrinsically generated action potentials, Purkinje cells were kept at -60 to -65 mV with hyperpolarizing current injections (<-250 pA). Under these conditions, MLI-PC IPSPs appeared as negative potentials ranging from -0.2 to -3 mV. Climbing fibers (CFs) were stimulated with a patch electrode filled with external solution located in the granule cell layer. To induce LTP of MLI-PC IPSPs (i.e., iLTP), a tetanus of five CF stimuli at 10 Hz was applied every 2 s for 3 min. For paired MLI-CF stimulation, each CF stimulus was coincided with two MLI stimuli, i.e., at 20 Hz. Purkinje cell holding current and input resistance were constantly monitored, and cells with >15% shift of these parameters during the recording were excluded from analysis.

### PURKINJE CELL SPONTANEOUS IPSCs AND REBOUND POTENTIATION

In a subset of recordings Purkinje cells were voltage clamped at -60 mV using intracellular solution containing (in mM): 150 CsCl, 15 CsOH, 1.5 MgCl_2_, 0.5 EGTA, 10 HEPES, 4 Na_2_ATP, and 0.4 Na_3_GTP (pH 7.3; osmolarity 300). Ten μM NBQX was supplemented in the ACSF to avoid contamination with spontaneous EPSCs. Spontaneous IPSCs were analyzed using Minianalysis (Synaptosoft, Decatur, USA). To analyze IPSC kinetics, unitary IPSCs of 50–100 pA were selected to avoid interference of noise or insufficient voltage clamp. Traces were scaled, averaged and fit using a single decay time constant. Series and input resistances were monitored every 3 min using hyperpolarizing voltage steps; recordings were terminated if the holding current or the series or input resistances changed >15%.

### PHARMACOLOGY

Baclofen (2 μM), cyclosporin A (5 μM), KN-93 (2 μM), SCH50911 (10 μM), KT 5720 (0.2 μM), and thapsigargin (10 μM) were obtained from Tocris Biosciences (Bristol, UK). Other chemicals were obtained from Sigma unless stated otherwise.

### STATISTICS

To test for statistically significant differences between wt and *Camk2a*^-^^/^^-^ and *Camk2b*^-^^/^^-^ recordings we used an unpaired, two-way Student’s *t*-test or a non-parametric Mann-Whitney *U* test depending on the distribution of the data. The level of significance (*p* < 0.05 or <0.001) is reported in the figure legends. To test whether a stimulus pattern induced a significant change we used a paired, two-way Student’s *t*-test on the last 5 min before the tetanus (pre-tetatnus) and the 20–25 min after the tetanus (post-tetanus). For these latter comparisons we considered *p*-values <0.05 to be significant.

## RESULTS

### BOTH α- AND βCa MKII SUBUNITS ARE ESSENTIAL FOR iLTP AT THE MLI-PC SYNAPSE

**FIGURE 1:**
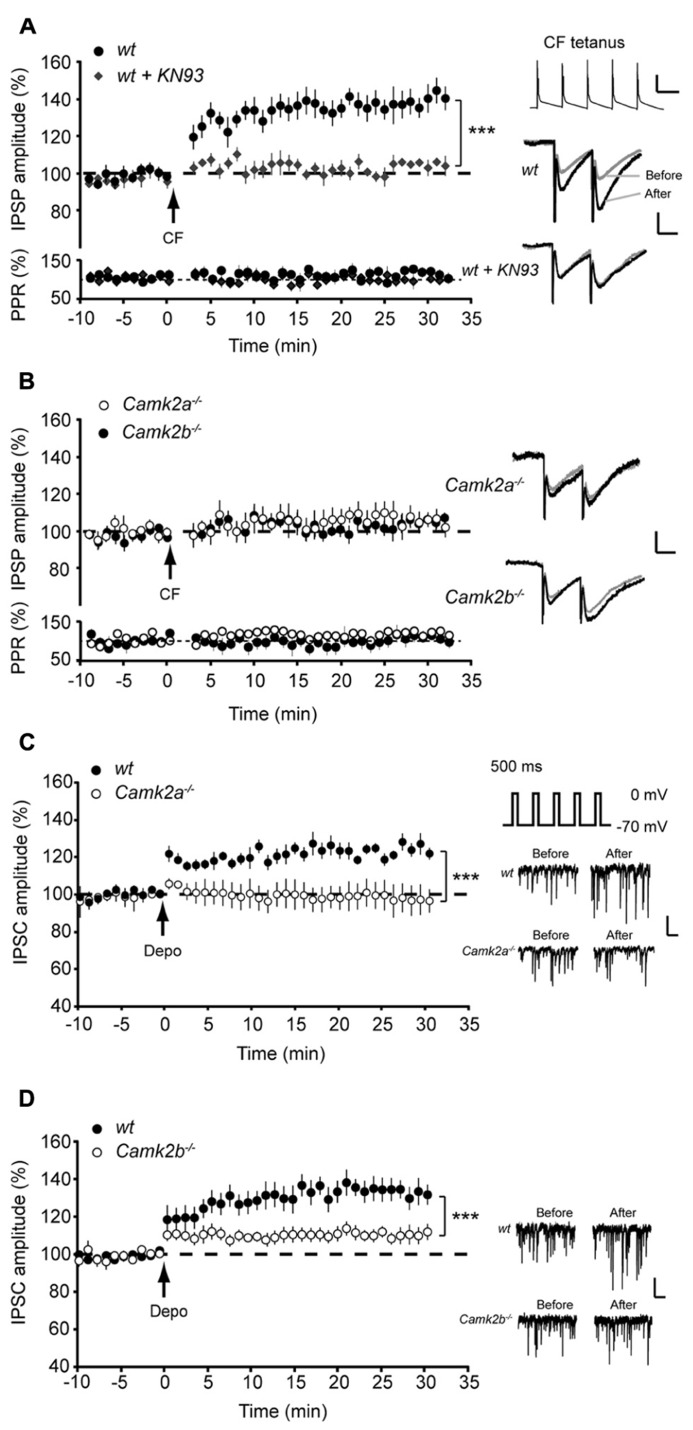
**Aberrant iLTP at MLI-Purkinje cell synapses in α and βCaMKII knockout mice.** (**A**; Top) Five pulses, 10 Hz climbing fiber (CF) stimulation repeated every 2 s for 3 min yields iLTP in wildtype (wt, *n* = 10) Purkinje cells but not in wt Purkinje cells in the presence of KN-93 (wt + KN93, *n* = 6). (Bottom) Accompanying paired pulse ratio of IPSPs. Inset: representative traces of CF stimulation, scale bars 20 mV/100 ms and representative traces of IPSP before and after the CF stimulation, scale bars 1 mV/25 ms. **(B)** CF stimulation did not induce iLTP in *Camk2a*^-^^/^^-^(*n* = 9) and *Camk2b*^-^^/^^-^ Purkinje cells (*n* = 8). **(C)** Inset shows schematic drawing of rebound potentiation experiment and representative traces of sIPSC, scale bars 50 pA/200 ms. Rebound potentiation in Purkinje cells was induced by five 500 ms depolarization pulses to 0 mV at 0.5 Hz; IPSC amplitudes were compared before and after tetanus. Impaired rebound potentiation in *Camk2a*^-^^/^^-^ mice (wt, *n* = 7; *Camk2a*^-^^/^^-^, *n* = 6). **(D)** Impaired rebound potentiation in *Camk2b*^-^^/^^-^ mice (wt, *n* = 8; *Camk2b*^-^^/^^-^, *n* = 9). Error bars represent SEM. Asterisks with brackets indicate statistical significance between wt and knockout mice (Student’s *t*-test of averages over last 5 min). ***p < 0.005.

To elucidate how α- and βCaMKII subunits mediate inhibitory synaptic plasticity, we investigated iLTP at MLI-PC synapses in *Camk2a*^-^^/^^-^ and *Camk2b*^-^^/^^-^ mice. To induce iLTP at MLI-PC synapses we activated the CF 5 times at 10 Hz every 2 s for 3 min (**Figure 1A**, inset); this tetanus significantly increased the MLI-IPSP amplitude in wild type (wt) Purkinje cells (averaged IPSP amplitude 20–25 min after the CF stimulus protocol (post-tetanus) was 138.2 ± 7.5% relative to the last 5 min pre-tetanus; *p* = 0.0002, **Figure 1A**). This iLTP occurred without inducing significant changes in the paired pulse ratio of two consecutive IPSPs with 50 ms interval (*p* = 0.18; **Figure 1A**), strongly suggesting that the site of plasticity was most likely postsynaptic. In accordance to previous reports that showed how the potentiation of inhibitory synaptic currents was fully blocked in wt by bath application of the global CaMKII blocker KN-93 (Kano et al., 1992; Kawaguchi and Hirano, 2000), our iLTP-induction protocol failed to induce a significant change in the postsynaptic responses to MLI stimulation (103.5 ± 2.1% relative to pre-tetanus; *p* = 0.18; **Figure 1A**). When the same CF stimulus protocol was delivered to either *Camk2a*^-^^/^^-^ or *Camk2b*^-^^/^^-^ Purkinje cells, we observed a significantly lower level of potentiation (108.8 ± 3.4 and 106.4 ± 2.5% of baseline IPSP amplitude, respectively; **Figure 1B**) than in wt Purkinje cells (*p* = 0.0005 and *p* = 0.0006; **Figures 1A,B**). Several possibilities could account for the reduction of iLTP induction in Purkinje cells of *Camk2a*^-^^/^^-^ or *Camk2b*^-^^/^^-^ mice. First, it is possible that deleting α- or βCaMKII induces a change in the surface level of GABA_A_-receptors and thus precludes the induction of iLTP in response to CF stimulation. This is unlikely, however, since the frequency, amplitude, and kinetics of spontaneously occurring (s)IPSCs in Purkinje cells were not significantly different between *Camk2a*^-^^/^^-^ and *Camk2b*^-^^/^^-^ mice and their wt littermates (**Table 1A**). It is also unlikely that the lack of iLTP originates from aberrant CF stimulation since none of the response parameters evoked by such stimulus, i.e., the Na^+^-spike, Ca^2^^+^-spike, and Ca^2^^+^-plateau amplitudes, was significantly different in *Camk2a*^-^^/^^-^ and *Camk2b*^-^^/^^-^ compared to their wt littermates (**Table 1B**). To test whether the lack of α- and βCaMKII prevents sufficient CF-stimulus induced Ca^2^^+^-influx to activate the molecular machinery underlying iLTP, we tested whether another trigger of Ca^2^^+^-influx could induce plasticity of spontaneously occurring IPSCs. Direct depolarization by voltage-clamping the Purkinje cell to 0 mV from a holding potential of -70 mV has been shown previously to effectively induce potentiation of sIPSCs in Purkinje cells (Kano et al., 1992). Five 500 ms depolarizing pulses from -70 to 0 mV with a 2 s interval readily potentiated sIPSCs in wt Purkinje cells, but not in *Camk2a*^-^^/^^-^ Purkinje cells (*p* = 0.002, **Figure 1C**). Similarly, the iLTP induced by depolarization was significantly reduced in *Camk2b*^-^^/^^-^ compared to wt (*p* = 0.001, **Figure 1D**). Together the effects of CF stimulation on MLI-IPSPs and of Purkinje cell depolarization on sIPSCs imply that both α- and βCaMKII are essential for post-synaptic iLTP at inhibitory synapses of Purkinje cells.

**Table 1:** Normal spontaneous IPSC properties in *Camk2a* ^-/-^ and *Camk2b* ^-/-^ Purkinje cells.

(A) sIPSC properties in *Camk2a* ^- / -^ and *Camk2b* ^- / -^ mice
	#	FF (Hz)	Amp (pA)	Rise (ms)	Decay (ms)	Width (ms)
wt	12	18.4 ± 1.7	62.8 ± 4.0	1.28 ± 0.1	15.1 ± 1.1	2.8 ± 0.2
*Camk2a*^-/-^	10	18.5 ± 0.8	65.1 ± 7.6	1.31 ± 0.1	13.7 ± 1.0	3.0 ± 0.2
*t*-tests		0.97	0.79	0.79	0.35	0.38
wt	13	19.2 ± 2.8	66.3 ± 3.0	1.0 ± 0.1	13.7 ± 0.9	2.6 ± 0.2
*Camk2b*^-/-^	12	19.0 ± 2.8	64.4 ± 2.5	1.0 ± 0.1	13.2 ± 0.6	2.6 ± 0.1
*t*-tests		0.97	0.64	0.63	0.61	0.97
**(B) Complex spike properties in *Camk2a*^- / -^ and *Camk2b*^- / -^ mice**				
	**#**	**Na** ^+^ spike (mV)		**1st Ca**^**2** +^ spike (mV)	**Ca**^**2** +^ plateau (mV)	**AHP (mV)**
wt	18	86.8 ± 4.8		56.8 ± 3.6	18.8 ± 1.0	-2.6 ± 0.2
*Camk2a*^-/-^	14	84.7 ± 2.9		51.4 ± 2.7	20.1 ± 1.7	-2.6 ± 0.2
*t*-tests		0.72		0.25	0.49	0.70
wt	16	85.8 ± 2.0		51.4 ± 1.8	21.6 ± 1.7	-2.6 ± 0.2
*Camk2b*^-/-^	15	81.5 ± 2.9		55.5 ± 1.6	21.2 ± 0.7	-2.3 ± 0.3
*t*-tests		0.22		0.10	0.65	0.43

***(A)** Normal spontaneous IPSC properties in *Camk2a*^-/-^ and *Camk2b*^-/-^ Purkinje cells. Table: quantification and comparison of spontaneous IPSC frequency (FF), amplitude (Amp), and kinetics (10–90% rise time, decay time constant and width at 50% of the maximal amplitude) of IPSCs. **(B)** Normal complex spike properties in *Camk2a*^-/-^ and *Camk2b*^-/-^ Purkinje cells. Table: quantification and comparison of the amplitudes of Na^+^ spike, the first Ca^2+^ spike (1st Ca^2+^ spike), the Ca^2+^ plateau, and after hyperpolarization (AHP) of the CF-EPSC in wt, *Camk2a*^-/-^ and *Camk2b*^-/-^ Purkinje cells.*

### BLOCKING PP2B ACTIVITY RESCUES iLTP IN *Camk*2b^-^^/^^-^ BUT NOT IN *Camk*2a^-^^/^^-^ MICE

**FIGURE 2:**
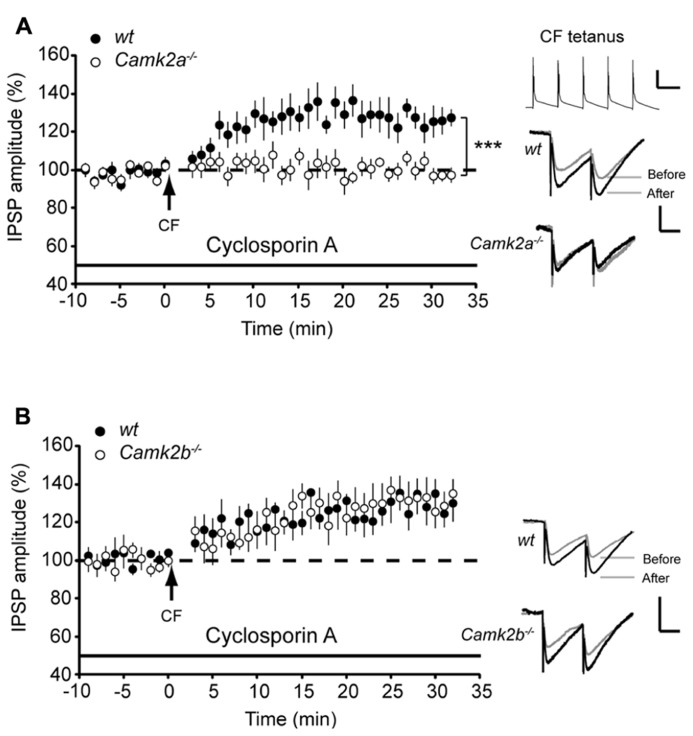
**Block of PP2B reveals iLTP and block of IP_**3**_ disrupts rescue of iLTP in *Camk2b*^-^^/^^-^ mice.** **(A)** CF stimulation yields iLTP in wt Purkinje cells (*n* = 7) in the presence of 5 μM PP2B inhibitor cyclosporin A, but not in *Camk2a*^-^^/^^-^ Purkinje cells (*n* = 10). Inset: representative traces of CF stimulation, scale bars 20 mV/100 ms and representative traces of IPSP before and after the CF stimulation, scale bars 1 mV/25 ms). (**B)** CF stimulation yields iLTP in wt Purkinje cells (*n* = 6) in the presence of 5 μM PP2B inhibitor cyclosporin A, as well as in *Camk2b*^-^^/^^-^ Purkinje cells (*n* = 8). Inset: representative traces of CF stimulation, scale bars 20 mV/100 ms and representative traces of IPSP before and after the CF stimulation, scale bars 1 mV/25 ms. Error bars represent SEM. Asterisks with brackets indicate statistical significance between wt and knockout mice (Student’s *t*-test of averages over last 5 min). ***p < 0.005.

Following direct post-synaptic depolarization or CF activity the calcium concentration rises in Purkinje cells, which activates not only α- and βCaMKII but also protein phosphatase 2B (PP2B), the latter of which counteracts the effects of CaMKII activation (Kawaguchi and Hirano, 2002; Belmeguenai and Hansel, 2005; van Woerden et al., 2009). In order to test whether the residual CaMKII in *Camk2a*^-^^/^^-^ and *Camk2b*^-^^/^^-^ mutants is outcompeted by PP2B we examined the effect of the specific PP2B blocker cyclosporin A on iLTP evoked by CF stimulation. Inhibiting PP2B activity did not alter the level of iLTP in wt and *Camk2a*^-^^/^^-^ Purkinje cells (129.9 ± 5.2 and 95.8 ± 4.9% compared to baseline IPSP amplitude, respectively; Figure 2A; *p* = 0.14 and 0.72 when compared to the condition without cyclosporin A as represented in **Figure 1A**). However, in *Camk2b*^-^^/^^-^ the presence of cyclosporin A the CF stimulus protocol resulted in a significant iLTP comparable to that recorded in the wt cells (129.3 ± 5.9 and 128.8 ± 5.2%, respectively; *p* = 0.95; **Figure 2B**). These data suggest that the residual αCaMKII in the *Camk2b*^-^^/^^-^, but not the residual βCaMKII in the *Camk2a*^-^^/^^-^mice, enables iLTP induction when PP2B is blocked.

### CO-ACTIVATION OF MLIs AND CF FACILITATES iLTP IN *Camk*2b^-^^/^^-^ BUT NOT IN *Camk*2a^-^^/^^-^ MICE

**FIGURE 3:**
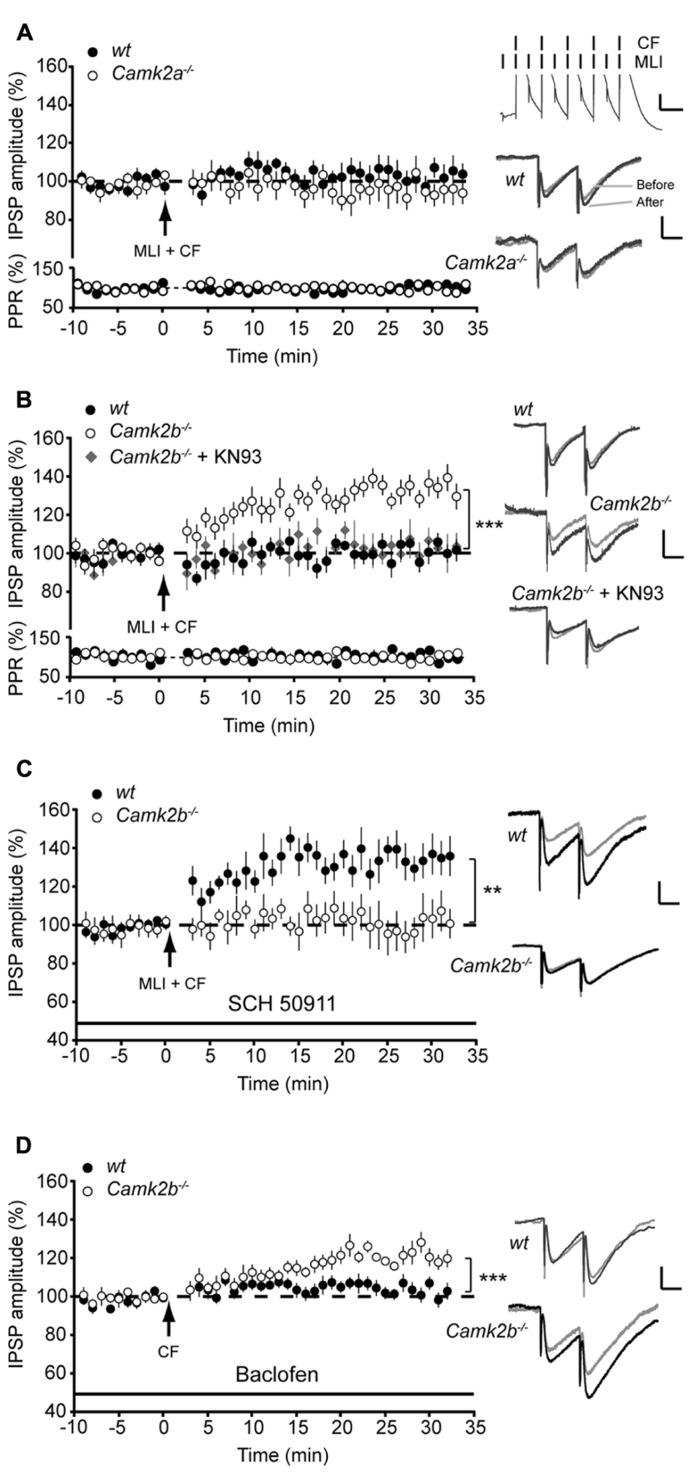
**GABA_**B**_-receptor activation facilitates iLTP in *Camk2b*^-^^/^^-^ but not in *Camk2a*^-^^/^^-^ mice.** (**A**; Top) Paired molecular layer interneuron (MLI; 20 Hz) and climbing fiber (CF; 5 pulses, 10 Hz) stimulation every 2 s for 3 min yields no change in IPSP amplitude in both wt (*n* = 10) and *Camk2a*^-^^/^^-^ (*n* = 8) Purkinje cells. (Bottom) Accompanying paired pulse ratio of IPSPs. Inset: representative traces of MLI-CF stimulation, scale bars 3 mV/100 ms and representative traces of IPSP before and after the CF stimulation, scale bars 1 mV/25 ms.**(B)** Paired MLI-CF stimulation yields no change in IPSP amplitude in wt Purkinje cells (*n* = 10) and in *Camk2b*^-^^/^^-^ Purkinje cells when KN93 is present (*Camk2b*^-^^/^^-^ + KN93, *n* = 5). MLI-CF stimulation significantly facilitates iLTP in *Camk2b*^-^^/^^-^ Purkinje cells (*n* = 13). **(C)** Inhibition of GABA_B_-receptor activation with SCH 50911 rescues iLTP in wt Purkinje cells (*n* = 9), but inhibits iLTP in *Camk2b*^-^^/^^-^ Purkinje cells (*n* = 7) following paired SC–CF stimulation. **(D)** Activation of GABA_B_-receptors with baclofen inhibits iLTP in wt Purkinje cells (*n* = 10), but facilitates iLTP in *Camk2b*^-^^/^^-^ cells (*n* = 8) following CF stimulation. Error bars represent SEM. Asterisks with brackets indicate statistical significance between wt and knockout mice (Student’s *t*-test of averages over last 5 min). **p < 0.01; ***p < 0.005.

Our results show that when PP2B activity is chemically blocked α- and βCaMKII serve differential roles during iLTP in Purkinje cells. One physiologically relevant cascade that mediates the impact of PP2B on iLTP at the MLI-PC synapse is controlled by activity of MLI’s during CF-stimulation (Kawaguchi and Hirano, 2000). Here we paired the five pulses of 10 Hz CF stimulation with 10 pulses of 20 Hz MLI stimulation (see inset **Figure 3A**). This paired stimulation suppressed iLTP at the MLI-PC synapses in both wt groups to levels not significantly different from *Camk2a*^-^^/^^-^ and *Camk2b*^-^^/^^-^ (103.6 ± 5.2%, *p* = 0.22 and 102.5 ± 3.7%, *p* = 0.52, respectively; **Figures 3A,B)**, without changing the paired pulse ratio (paired Student’s *t*-test of averages pre- vs. post-tetanus: all *p*-values > 0.7). In *Camk2a*^-^^/^^-^ this suppression protocol did not induce a significant change in synaptic strength (97.8 ± 4.1% of the pre-tetanus IPSP amplitude; *p* = 0.29; **Figure 3A**), whereas in *Camk2b*^-^^/^^-^ the same conditions evoked iLTP (132.2 ± 4.8%; *p* = 0.0002; **Figure 3B**). We next tested whether this unexpected expression of iLTP in *Camk2b*^-^^/^^-^ mice in response to a stimulation protocol that suppresses iLTP in wt mice was dependent on the activity of residual αCaMKII. We repeated the paired MLI-CF protocol in the presence of KN-93 in *Camk2b*^-^^/^^-^ mice. Indeed, in this condition, i.e., when all residual CaMKII activity is blocked, no detectable iLTP was found in *Camk2b*^-^^/^^-^ mice (103.0 ± 5.4% of the pre-tetanus IPSP amplitude; *p* = 0.60; **Figure 3B**). These results indicate that in the absence of βCaMKII MLI-CF stimulation induced iLTP by activation of residual αCaMKII.

### GABA_B_-RECEPTOR ACTIVATION FACILITATES iLTP IN *Camk*2b^-^^/^^-^ BUT NOT IN *Camk*2a^-^^/^^-^ MICE

The molecular mechanism underlying the effect of MLI stimulation on Ca^2^^+^- and CaMKII-dependent potentiation of inhibitory responses in Purkinje cells have been linked to GABA_B_-receptor activation (Kawaguchi and Hirano, 2000; Kawaguchi and Hirano, 2002). To study whether the GABA_B_-receptor activation is essential for iLTP, we next blocked the GABA_B_-receptor activation with SCH 50911 during the paired MLI-CF stimulation protocol. Under these conditions the MLI-CF stimulation did not evoke iLTP in *Camk2b*^-^^/^^-^(101.7 ± 8.9% of the pre-tetanus IPSP amplitude; *p* = 0.69; **Figure 3C**). The efficacy of this approach is indicated by the fact that in wt SCH 50911 cancelled the suppression effect of co-activating the MLI-CF inputs, i.e., iLTP could be induced (133.3 ± 8.4% of the pre-tetanus IPSP amplitude; *p* = 0.002; **Figure 3C**). To study whether GABA_B_-receptor activation paired with CF stimulation is also sufficient to evoke iLTP in *Camk2b*^-^^/^^-^ mutants we replaced the MLI stimulation with the bath-applied GABA_B_-receptor agonist Baclofen. In the presence of Baclofen CF stimulation evoked iLTP in *Camk2b*^-^^/^^-^ (121.0 ± 4.1% of the pre-tetanus IPSP amplitude; *p* = 0.002) and suppressed iLTP in wt (101.5 ± 2.7%; *p* = 0.61; **Figure 3D**). Together these experiments unequivocally show that in wt GABA_B_-receptor activation suppresses CF-evoked iLTP, but that in *Camk2b*^-^^/^^-^ GABA_B_-receptor activation is both essential but also sufficient to facilitate iLTP evoked by CF-activity.

### CaMKII SUBUNITS MAY DIFFERENTIATE THE EFFECTS OF GABA_**B**_-RECEPTORS ON iLTP

**FIGURE 4:**
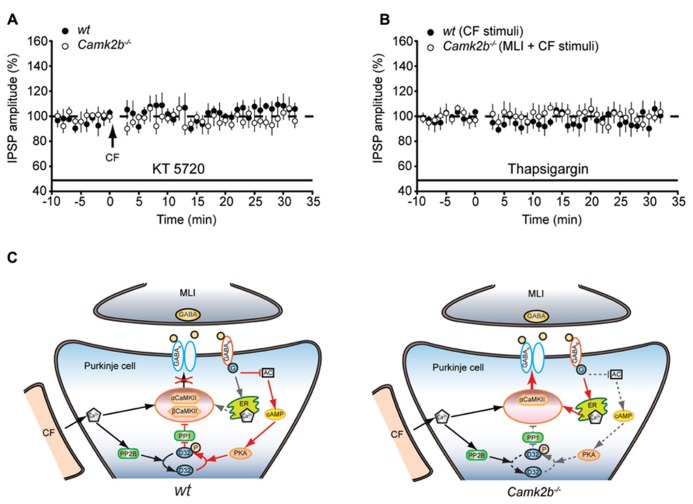
**GABA_B_-receptor activationmay operate two distinct pathways to activate or inhibit the iLTP induction.** **(A)** Left, schematic representation of the working model of CaMKII mediated iLTP induction cascade and GABA_B_-receptor mediated inhibition of iLTP in wt Purkinje cell. The model is proposed based on previous studies (Kano et al., 1996; Kawaguchi and Hirano, 2002) and the current data. Cascades are simplified for the clarity of illustration. Arrows indicate activation cascades, bars indicate inhibitory cascades. Note that in the presence of both α and βCaMKII, the calcium release from internal stores upon GABA_B_-receptor activation is outcompeted by the suppressing PKA-PP1 pathway (dashed arrow). AC, adenylyl cyclase; D32, DARPP-32. Right, schematic representation of the CaMKII mediated iLTP induction cascade and GABA_B_-receptor mediated inhibition of iLTP in *Camk2b*^-^^/^^-^ Purkinje cells. Genetic deletion of βCaMKII revealed a rescue of iLTP by GABA_B_-receptor activation. Note that (1) the inhibitory effect of PKA-PP1 pathway upon GABA_B_-receptor activation is minimized (indicated in dashed lines) in the absence βCaMKII and that (2) the facilitating effects of calcium release from internal stores enables the rescue of iLTP. **(B)** Inhibition of PKA with KT5720 suppresses iLTP in wt Purkinje cells (*n* = 5), but does not rescue iLTP in *Camk2b*^-^^/^^-^Purkinje cells (*n* = 6) following CF stimulation. **(C)** Inhibition of calcium release from internal stores with thapsigargin abolishes the facilitation of iLTP in *Camk2b*^-^^/^^-^ Purkinje cells (*n* = 7) following paired MLI-CF stimulation, as well as iLTP in wt Purkinje cells (*n* = 6) following CF stimulation. Error bars represent SEM. Asterisks with brackets indicate statistical significance between wt and knockout mice.

How can GABA_B_-receptor activation inhibit iLTP in wt and facilitate iLTP in *Camk2b*^-^^/^^-^ mice? It is known that GABA_B_-receptor activation mediates the activity of two separate pathways (**Figure 4A**); upon GABA_B_-receptor activation protein kinase A (PKA) is inhibited, which promotes the PP2B-dependent suppression of the CaMKII-mediated iLTP (Kawaguchi and Hirano, 2002); and GABA_B_-receptor activation induces calcium release from internal stores, which could promote iLTP (Komatsu, 1996; Yamauchi, 2005). We hypothesized that the presence of βCaMKII determines which of these pathways prevails and thereby whether upon MLI-CF co-activation iLTP is induced or not. To test this working hypothesis, we first assessed whether GABA_B_-mediated inhibition of PKA, which is critical for the suppression of iLTP in wt (Kawaguchi and Hirano, 2002), also mediates the rescue of iLTP in *Camk2b*^-^^/^^-^. However, the presence of the PKA blocker KT5720 did not result in a rescue of iLTP in *Camk2b*^-^^/^^-^ following CF stimulus (97.4 ± 7.0% of the pre-tetanus IPSP amplitude; *p* = 0.61) whereas it did block iLTP in wt Purkinje cells [106.22 ± 0.83%; *p* = 0.68; **Figure 4B**; see also (Kawaguchi and Hirano, 2002)]. These results indeed indicate that GABA_B_-receptor activation facilitates iLTP in *Camk2b*^-^^/^^-^ by a separate pathway that is PKA-independent. To test whether instead of the PKA-pathway the GABA_B_-mediated calcium release from internal stores controls the rescue of iLTP in *Camk2b*^-^^/^^-^ evoked by the suppression protocol, we tested the effect of thapsigargin, which depletes intracellular calcium stores. In the presence of thapsigargin, the suppression protocol failed to rescue iLTP in *Camk2b*^-^^/^^-^ (94.6 ± 3.0% of the pre-tetanus IPSP amplitude; *p* = 0.74) and the CF protocol failed to induce iLTP in wt (99.9 ± 5.7%; *p* = 0.88; **Figure 4C**). Together these results support our working hypothesis that GABA_B_-receptor activation suppresses iLTP in the presence of βCaMKII in a PKA-dependent manner, but rescues iLTP in the absence of βCaMKII by raising the intracellular calcium concentration through calcium release from internal stores.

## DISCUSSION

The current study shows that αCaMKII and βCaMKII both play a role in induction of iLTP at MLI-PC synapses, but that both isoforms can be activated selectively and serve a distinct function in this process. Two lines of evidence support these conclusions. First, when the competing PP2B activity is blocked, iLTP is only expressed following CF stimulation in *Camk2b*^-^^/^^-^, not in the *Camk2a*^-^^/^^-^. Second, whereas co-activation of MLIs and CF suppresses iLTP in wt, this protocol permits iLTP induction in *Camk2b*^-^^/^^-^. Thereby our results indicate that the presence of βCaMKII determines whether activation of αCaMKII evokes iLTP at MLI-PC synapses.

Several studies confirmed the involvement of the CaMKII holo-enzyme in synaptic plasticity at inhibitory synapses in the hippocampus, amygdala, cerebral cortex, and cerebellum (Kano et al., 1996; Kawaguchi and Hirano, 2002; Bauer and LeDoux, 2004; Maffei et al., 2006; Xu et al., 2008; Houston et al., 2009; Castillo et al., 2011), but it has not yet been possible to decipher the individual contributions of α- and βCaMKII to iLTP. Given the overlap of molecular components between the signaling pathways that control synaptic plasticity at both excitatory and inhibitory synapses (Collingridge et al., 2004), one would predict distinct roles of α- and βCaMKII in iLTP much alike those recently described for excitatory synapses (Cho et al., 2007; van Woerden et al., 2009). Indeed, we found that in *Camk2b*^-^^/^^-^ Purkinje cells the GABA_B_-activation was essential to elicit iLTP upon CF stimulation, whereas in *Camk2a*^-^^/^^-^ Purkinje cells GABA_B_-activation did not trigger iLTP. Due to the impact of blocking calcium release on the expression of iLTP in *Camk2b*^-^^/^^-^ these results seem in accordance with the predicted lower calcium sensitivity of αCaMKII in *Camk2b*^-^^/^^-^ Purkinje cells than in wt *Camk2a*^-^^/^^-^ Purkinje cells (Brocke et al., 1999). However, the fact that the same stimulus protocol can suppress iLTP in Purkinje cells when βCaMKII molecules are present argues against a dominant role of the enhanced calcium sensitivity of βCaMKII. An alternative possibility is that the actin-binding domain of βCaMKII may act as a differentiator: in *Camk2b*^-^^/^^-^ mutants the residual αCaMKII is not confined to actin and thereby can be more readily activated by local calcium sources like intracellular calcium stores (Finch and Augustine, 1998), store-operated calcium influx, or activation of the transient receptor potential canonical (TPRC) channels, all of which promote CaMKII-mediated iLTP (Shen et al., 1998; Hirono et al., 2001; New et al., 2006; Xu et al., 2008; Chae et al., 2012). This hypothesis should be tested in future experiments, taking into account that any of these local calcium sources may be essential for iLTP induction as well (Komatsu, 1996).

Although our study focussed on the post-synaptic effects of the absence of either αCaMKII or βCaMKII, we cannot exclude the possibility that a presynaptic function of CaMKII, such as phosphorylating synapsin-1 and thereby enhancing neurotransmitter release, is also affected. Yet, our recordings on the spontaneous release and stimulus-evoked GABA release from MLI terminals and glutamate release from CF terminals in *Camk2a*^-^^/^^-^ and *Camk2b*^-^^/^^-^ does not show any significant difference (**Table 1**). Still, MLIs as well as neurons in the inferior olive express βCaMKII (but not αCaMKII; Hansel et al., 2006) and could therefore in principle be subject to affected neurotransmitter release in *Camk2b*^-^^/^^-^. Since several studies have shown a role of CaMKII in neurotransmitter release from other cerebellar neurons such as granule cells (e.g., León et al., 2008), a more detailed study on the presynaptic effects of the lack of βCaMKII that focusses on the release probability in MLIs and CFs is warranted.

The induction rules for plasticity of inhibitory synapses at cerebellar MLI-PC synapses are opposite to those in early post-natal hippocampal CA1 tissue: coincident pre- and postsynaptic activity results in suppression of iLTP at MLI-PC synapses through GABA_B_-receptor activation (Kawaguchi and Hirano, 2000), whereas this cascade is essential for iLTP at CA1 synapses (Xu et al., 2008). Our data show that genetic ablation of βCaMKII reverts the iLTP induction rules at cerebellar MLI-PC synapses to hippocampal CA1-like rules, in that coincident presynaptic activity is essential for the induction of iLTP in *Camk2b*^-^^/^^-^. This surprising finding at this inhibitory synapse shows a remarkable coherence with the inversion of induction rules of long-term plasticity at excitatory parallel fiber – Purkinje cell synapses (van Woerden et al., 2009). Here too, the lack of βCaMKII reversed the induction rules for LTP and LTD, highlighting the overlap in molecular pathways of inhibitory and excitatory plasticity (Collingridge et al., 2004). Moreover, recent evidence indicates that local calcium concentrations control the selective translocation of αCaMKII molecules to either excitatory or inhibitory synapses in hippocampal tissue (Marsden et al., 2010), physically merging the molecular pathways that control plasticity at both types of synapses. Current studies promote a central role of βCaMKII in coordinating the translocation of CaMKII holo-enzyme complexes in excitatory synapses in cerebellar Purkinje cells (van Woerden et al., 2009). Here, we have provided evidence that βCaMKII may also play a similar pivotal role at its inhibitory synapses.

## Conflict of Interest Statement

The authors declare that the research was conducted in the absence of any commercial or financial relationships that could be construed as a potential conflict of interest.

## AUTHOR CONTRIBUTIONS

Zhenyu Gao and Freek E. Hoebeek were involved in the conception and design of the experiments, data collection, analysis, and interpretation. All authors were involved in drafting and critical commenting on the manuscript. All authors approved this manuscript.

## Abbreviations

AC: adenylyl cyclase
CaMKII: Calcium/Calmodulin-dependent kinase type II
CF: Climbing fiber
D-AP5: D(-)-2-amino-5-phosphonopentanoic acid
GABA: γ-aminobutyric acid
iLTP: inhibitory long-term potentiation
IP3: inositol-tri-phosphate
IPSC: inhibitory postsynaptic current
IPSP: inhibitory postsynaptic potential
LTP: long-term potentiation
MLI-PC: molecular layer interneuron – Purkinje cell
NBQX: 2,3-dihydroxy-6-nitro-7-sulfamoyl-benzoquinoxaline
PKA: protein kinase A
PP1: Phosphoprotein phosphatase 1
PP2B: protein-phosphatase type IIB
PTX: picrotoxin
TPRC: transient receptor potential canonical
